# Molecular epidemiology of Aleutian mink disease virus from fecal swab of mink in northeast China

**DOI:** 10.1186/s12866-020-01910-8

**Published:** 2020-08-01

**Authors:** Mingwei Tong, Na Sun, Zhigang Cao, Yuening Cheng, Miao Zhang, Shipeng Cheng, Li Yi

**Affiliations:** 1 School of Basic Medical Sciences, Shanxi Medical University, Taiyuan, Shanxi province P. R. China; 2 Institute of Special Wild Economic Animal and Plant Science, Chinese Academy of Agricultural Sciences, 4899 Juye Street, Changchun, 130122 Jilin Province P. R. China

**Keywords:** Aleutian mink disease parvovirus, Molecular epidemiology, Hypervariable region, Fecal swab, VP2 gene

## Abstract

**Background:**

Aleutian mink disease parvovirus (AMDV) causes Aleutian mink disease (AMD), which is a serious infectious disease of mink. The aim of this study was to get a better understanding of the molecular epidemiology of AMDV in northeast China to control and prevent AMD from further spreading. This study for the first time isolated AMDV from fecal swab samples of mink in China.

**Results:**

A total of 157/291 (54.0%) of the fecal swab samples were positive for AMDV. Of these, 23 AMDV positive samples were randomly selected for sequence alignment and phylogenetic analysis based on the acquired partial fragments of VP2 gene with the hypervariable region. Comparative DNA sequence analysis of 23 AMDV isolates with a reference nonpathogenic (AMDV-G) strain revealed 8.3% difference in partial VP2 nucleotide sequences. Amino acid alignment indicated the presence of several genetic variants, as well as one single amino acid residue deletion. The most concentrated area of variation was located in the hypervariable region of VP2 protein. According to phylogenetic analysis, the Chinese AMDV strains and the other reference AMDV strains from different countries clustered into three groups (clades A, B and C). Most of the newly sequenced strains were found to form a Chinese-specific group, which solely consisted of Chinese AMDV strains.

**Conclusion:**

These findings indicated that a high genetic diversity was found in Chinese AMDV strains and the virus distribution were not dependent on geographical origin. Both local and imported AMDV positive species were prevalent in the Chinese mink farming population. The genetic evidence of AMDV variety and epidemic isolates have importance in mink farming practice.

## Background

Aleutian mink disease (AMD) caused by Aleutian mink disease parvovirus (AMDV) is the most commercially important infectious disease affecting farmed mink (*Neovison vison*) worldwide. It causes a considerable economic loss to the mink farming industry by reducing reproductive output and decreasing the fur value. Clinical signs of progressive AMD infection in adult mink include the excessive level of plasma and gamma globulins in blood, inflammation of the glomeruli in kidney, decreased fertility, spontaneous abortion, and severe chronic immune dysfunction [1, 2].

AMDV is a member of the Parvovirus family [3]. It has an approximately 4.8 kb linear single-stranded genome, which can encode two structural proteins (VP1 and VP2) and three non-structural proteins (NS1, NS2 and putative NS3) [4, 5]. The VP2 protein is a central capsid protein, and plays an important role in the pathogenicity and host range of the AMDV, as AMDV strains show a high degree of variability in VP2 gene, especially in the hypervariable region [6, 7]. AMDV isolates can be distinguished by the detection of the hypervariable region in VP2 gene, and the definition of the hypervariable region contributes to preliminary typing [8]. Therefore, the study on the hypervariable region of VP2 gene is of great interest for AMDV identification and vaccine development.

At present, only a few molecular epidemiological reports of AMDV isolated from tissue or blood have been studied [9–11]. Since AMD infection can be transmitted in the mink farming industry through feces, fecal samples are feasible for detecting AMDV. In previous report, the fecal swabs have been used for AMDV virus isolation and detection of AMDV infection in farm in Spain [12].

In northeast China, Jilin, Liaoning and Heilongjiang provinces are known as the main mink breeding area. The majority of the farmed mink species is indigenous to China or primarily imported from Denmark and the USA [6]. To obtain a better understanding of the genetic diversity, relationships of different lineages of AMDV and molecular epidemiology of AMDV in northeast China, the fecal swabs samples were collected from major mink farming areas and used to investigate the origins of AMDV infection.

## Results

### Detection of AMDV

A total of 157 samples from three different farms were detected by PCR, showing an AMDV-positive rate of 54.0%. Among these, about 45.9% (89/194) and 70.1% (68/97) of the mink samples were AMDV-positive in Jilin province and Dalian city of Liaoning province, respectively (Table 1).

**Table 1:** PCR detection results regarding AMDV infection in samples from three farms

Farm	Region	Positive	Negative	Total	Positive ratio (%)
I	China, Jilin	49	45	94	52.1
II	China, Jilin	40	60	100	40
III	China, Dalian	68	29	97	70.1
	Total	157	134	291	54.0

### Nucleotide sequence and amino acid sequence analyses

A total of 23 AMDV-positive mink samples were randomly selected from three farms for sequence analysis. The acquired partial fragments of VP2 nucleotide sequences from 23 AMDV strains were compared with the corresponding sequence of nonpathogenic AMDV-G strain. The results showed that the detected 23 partial fragments of VP2 DNA sequences had 92.2–99.3% identity to each other, and 91.7–95.0% identity to AMDV-G strain. Comparing our identified AMDV sequences with AMDV-G strain revealed up to 8.3% difference between tested partial fragments of nucleotide sequences in the VP2 gene.

**Fig. 1:**
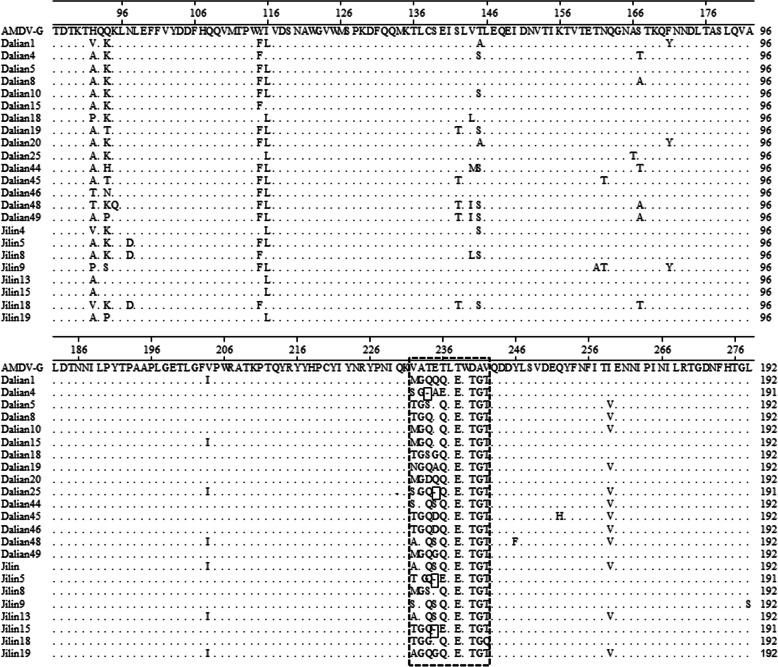
Alignment of the deduced amino acid sequences predicted from partial VP2 gene fragments from AMDV-G strain and acquired Chinese AMDV isolates in this research. Amino acids with the same as AMDV-G sequence are shown by dots. The numbers at top represent the amino acid positions of AMDV-G VP2 protein. represents the acid residue deletions. indicates the hypervariable region

The alignment was built using the deduced amino acid sequences (192 aa) predicted from the detected fragments of VP2 sequences from 23 newly sequenced AMDV strains and AMDV-G strain. Several variants among the 192 amino acid residues were observed in Fig. 1. Approximately 28 amino acid residue differences were identified, including one single amino acid (Gln) residue deletion in some isolates (Fig. 1). Of these, 9 different residues were found in a 11-amino-acid region (aa 232–242), corresponding to the hypervariable region of the VP2 gene.

### Phylogenetic analysis

**Fig. 2:**
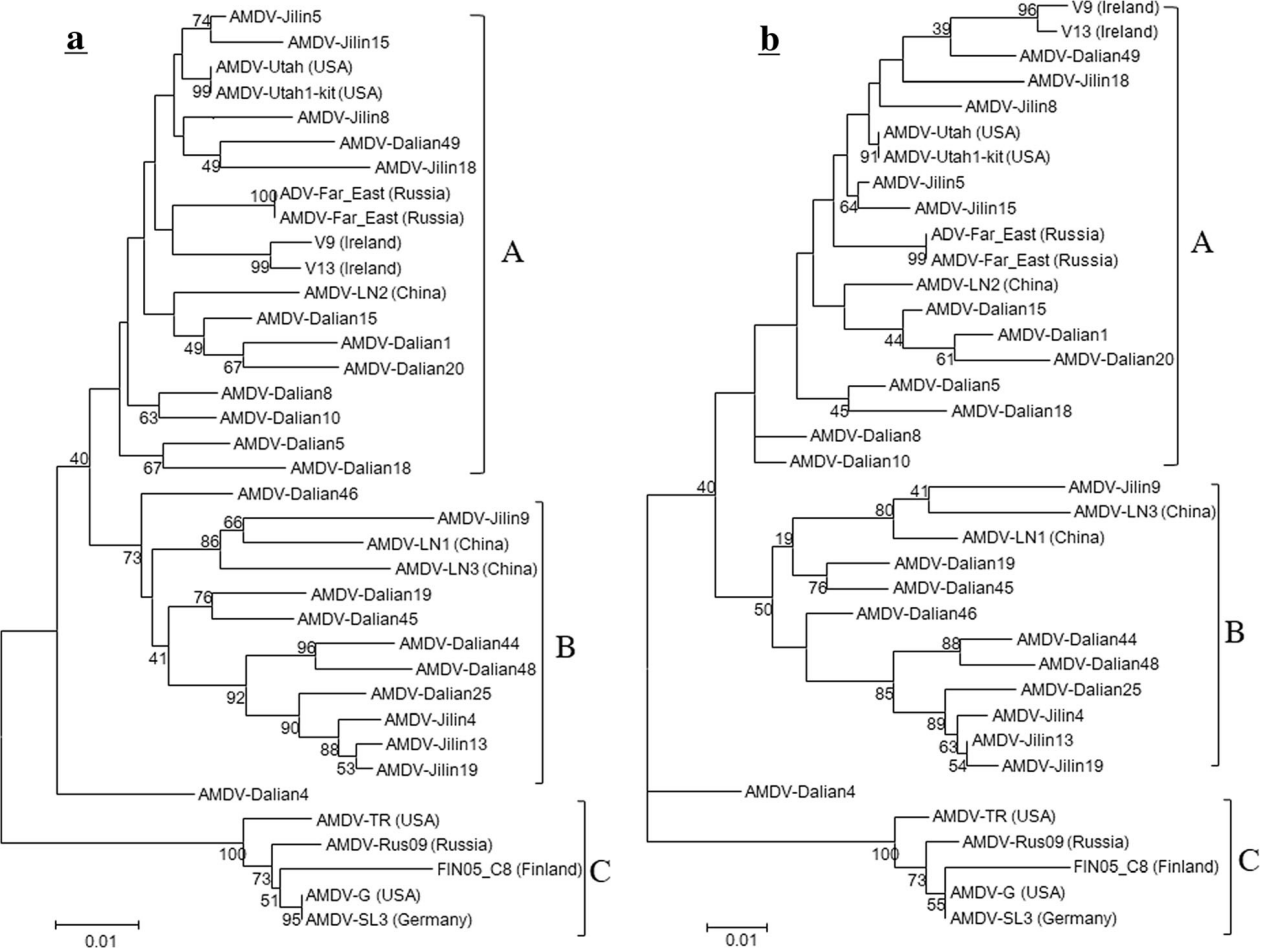
Phylogenetic tree of 37 AMDV isolates based on alignment of the partial fragments of VP2 gene. The tree was constructed using neighbor-joining method (**a**) and maximum likelihood method (**b**). Bootstrap values higher than 40% (**a**) or 39% (**b**) are shown (1000 replications). The reference sequences were obtained from the GenBank database, and are marked by the country origins (China, USA, Russia, Germany, Finland and Ireland). The strains were separated into three groups designated by the letters **A**, **B** and **C**. Scale bar indicates the average number of amino acids substitutions per site

To identify the overall possible evolutionary relationships of AMDV in northeast China, a phylogenetic tree based on 37 detected fragments of VP2 sequences was constructed, including 23 newly sequenced Chinese strains and 14 reference strains from different countries. According to the tree topology, AMDV isolates formed three major groups (Fig. 2A). At present, since no official genotyping is available for the AMDV VP2 gene [5], the gene groups were designated as clades A, B, and C.

The Chinese AMDV sequences were distributed in all three groups (Fig. 2A). Clade A consisted of AMDV isolates from across China, the USA, Russia and Ireland. As shown in clade A, AMDV-Jilin5/8/15/18 and AMDV-Dalian 49 isolates were closer to AMDV-Utah and AMDV-Utah1-kit strains, and AMDV-Dalian1/15/20 strains were closely related to LN-2 isolate, indicating that these strains shared a common ancestor. Clade B contained isolates solely from China, suggesting a close relationship of these strains with AMDV-LN1 and AMDV-LN3. Clade C consisted primarily of AMDV isolates from foreign countries as well as one single Chinese isolate (Dalian 4) (Fig. 2a). Maximum likelihood (Fig. 2b) and maximum parsimony (Fig. S1) phylogenetic analyses also formed three main groups as seen in the neighbor-joining phylogenetic tree, being a little bit different in maximum parsimony phylogenetic tree. Dalian 1, Dalian 15, Dalian 20 and LN2 isolates were clustered in clade A by neighbor-joining and maximum likelihood analyses, which seemed more likely to cluster in a single clade by phylogenetic analysis using maximum parsimony method (Fig. S1).

## Discussion

Chinese mink population has become popular in the world at present due to the introduction and propagation of mink in the late 1950s for the fur industry in China. So far, AMD has severely affected farmed mink in China as well as worldwide. Currently, there is no available vaccine, and the knowledge about AMDV diversity and molecular epidemiology will help to prevent AMD from spreading.

Previous studies reported that a large number of mink farms were affected by AMDV in China, based on VP2 or NS1 gene from samples of mink tissues, such as spleen, and plasma, especially during the pelting season [9, 10]. However, this epidemiological survey of AMDV is commonly limited by sampling time and quantity. Fecal swab sampling is easy to perform in a large population and causes small or no damage to animals. In this study, the fecal swabs samples were used to investigate the prevalence of AMDV in the major mink farming areas in northeast China, and the high prevalence (54.0%) of AMDV was found.

Nucleotide sequence comparisons of acquired fragments of the VP2 gene revealed that AMDV isolates from different farmed minks in northeast China were closely related to each other, but different from the nonpathogenic AMDV-G strain. There are 28 amino acid differences in amino acid alignment, which were distributed in all segments of the detected genes. Interestingly, of these amino acids, 9 amino acids were centrally present in the hypervariable region. Morever, 23 Chinese samples had similar changes in some fixed positions, especially in the hypervariable regions. These results indicated that Chinese AMDV strains had a high degree of similarity among themselves, while they showed the difference from ADMV strains in other countries, which is consistent with the previous studies [9, 13].

The neighbor-joining method was used to construct a phylogenetic tree. However, due to the low confidence of some branches (possibly due to the short sequences used), maximum likelihood and maximum parsimony methods were also performed to verify the reliability of the neighbor-joining phylogenetic tree. The trees constructed by maximum likelihood and maximum parsimony methods were the same or quite similar as showed in neighbor-joining phylogeny, and the bootstrap trees were relatively consistent, which enhance the reliability of neighbor-joining analysis [14].

All the Dalian samples were collected from one farm of Liaoning province. The phylogenetic analysis found that AMDV-Dalian isolates appeared in all three groups, which showed that different AMDV genotypes could coexist in the same farm. Besides, AMDV-Jilin isolates clustered in clades A and B, with the same as many of the AMDV-Dalian isolates (except for Dalian 4 isolate), revealing similar AMDV genotypic distribution in different farms from Jilin and Liaoning provinces, which further indicated that the distribution of AMDV strains was not related to the geographical origin. The AMDV distribution in northeast China probably results from the frequent mink trade among different farms and the introduction of both domestic and foreign minks, which contributes to improving local mink availability, but simultaneously leads to the enhancive virus spreading.

The phylogenetic analysis also revealed a regional-specific clade B, which only contained the isolates from China, and the clusters of the Chinese AMDV strains may reflect a long history in the breeding of domestic minks. Furthermore, the Chinese AMDV isolates were distributed in all three groups, indicating that the newly isolated Chinese AMDV strains consisted of both prevalent indigenous strains and isolates imported from abroad, which further implied that both local and imported AMDV species were prevalent in the farmed Chinese minks. Similar findings were found by previous reports [6, 10].

## Conclusion

In summary, AMD causes severe economic loss to the fur farming industry. Molecular epidemiological studies are necessary to assess patterns of disease transmission to improve disease detection methods. Our study is the first to report AMDV infection rates using fecal swap samples in China. The evidence of AMDV variety and epidemic isolates have importance in mink farming practice. The present report lays a good foundation for further study of AMDV in China.

## Methods

### Sample collection and preparation

During August–November 2014, a total of 291 fecal swabs samples from minks were obtained from three different farms in Jilin and Liaoning provinces in northeast China. The 94 and 100 samples were collected from farms I and II of Jilin province, respectively. A total of 97 samples were obtained from farm III of the Dalian city of Liaoning province. During sample collection, precautions were taken to avoid cross-contamination of the samples. All samples were stored at − 80 °C for further analysis.

### DNA extraction and sequencing

Genomic DNA of AMDV was extracted by an EasyPure Genomic DNA kit (TransGen Biotech Inc., Beijing, China) according to the manufacturer’s instructions. A pair of PCR primers was designed based on the previous reports [8, 15, 16] to amplify partial nucleotide fragments covering the hypervariable region of AMDV VP2 gene. The primers were as follows: Forward: 5′-ACTTGATATTTAATGCTGGTAGAA-3′ (2596–2619 nt), and Reverse: 5′-CATGGTTTACTTTTAAACTCA-3′ (3200–3220 nt). The expected PCR product was edited to a length of 625-bp corresponding to the nucleotide position (2596–3220) of ADV-LN2 (Accession no. GU183265.1) or the amino acid position (82–285) of VP2 protein.

PCR amplification was performed in a 25-μL reaction mixture containing 5 μL of template DNA, 1 × PCR buffer, 0.25 mM of dNTP, 0.5 μM of each primer, and 1 unit of Taq polymerase (TransGen Biotech Inc., Beijing, China). The PCR conditions were as follows: 94 °C for 5 min, 35 cycles of 94 °C for 30 s, 55 °C for 1 min, and 72 °C for 1 min, and a final extension step at 72 °C for 7 min. Positive (DNA extracted from previously preserved AMDV strain in our laboratory) and blank controls (water) were used in each set of amplification.

The amplified products (25 μL) were visualized after staining with ethidium bromide and separated by electrophoresis on 1.0% agarose gels. The PCR products were purified with an EasyPure Gel Extraction Kit (TransGen Biotech Inc., Beijing, China), and then cloned into pEASY-T1 vector and transformed into Trans1-T1 Phage Resistant Chemically Competent Cells (TransGen Biotech Inc., Beijing, China). The positive clones were sequenced at Invitrogen, Shanghai, China. The sequences were edited using DNAStar software.

### Genetic analysis

The partial fragments of VP2 nucleotide and deduced amino acid sequences of our newly sequenced AMDV isolates were assembled and aligned by BioEdit 7.0.5.3 [8] and MegAlign. The nonpathogenic AMDV-G strain (Accession no.: M20036) was used as the reference sequence.

To reveal a possible evolutionary trend of AMDV, a phylogenetic tree was constructed based on the comparison of our 23 partial VP2 nucleotide sequences with the corresponding sequences of 14 reference strains from China, USA, Russia, Germany, Finland and Ireland. The phylogenetic trees were constructed by the neighbor-joining method, using the bootstrap method with 1000 replicates. The maximum likelihood method and maximum parsimony method were also performed for phylogenetic analysis to assess the reliability of the neighbor-joining phylogenetic analysis. The AMDV strains used in this study were shown in Table 2.

**Table 2:** Details of the AMDV strains used in sequence analysis and phylogenetic analysis reported in this study

Strain	Isolation year	Country and region	Origin	Reference	GenBank accession no.
AMDV-Dalian1	2014	China, Liaoning	China	This publication	KT257635
AMDV-Dalian4	2014	China, Liaoning	China	This publication	KT257649
AMDV-Dalian5	2014	China, Liaoning	China	This publication	KT257650
AMDV-Dalian8	2014	China, Liaoning	China	This publication	KT257651
AMDV-Dalian10	2014	China, Liaoning	China	This publication	KT257634
AMDV-Dalian15	2014	China, Liaoning	China	This publication	KT257645
AMDV-Dalian18	2014	China, Liaoning	China	This publication	KT257646
AMDV-Dalian19	2014	China, Liaoning	China	This publication	KT257647
AMDV-Dalian20	2014	China, Liaoning	China	This publication	KT257636
AMDV-Dalian25	2014	China, Liaoning	China	This publication	KT257648
AMDV-Dalian44	2014	China, Liaoning	China	This publication	KT257652
AMDV-Dalian45	2014	China, Liaoning	China	This publication	KT257653
AMDV-Dalian46	2014	China, Liaoning	China	This publication	KT257654
AMDV-Dalian48	2014	China, Liaoning	China	This publication	KT257655
AMDV-Dalian49	2014	China, Liaoning	China	This publication	KT257656
AMDV-Jilin4	2014	China, Jilin	China	This publication	KT257641
AMDV-Jilin5	2014	China, Jilin	China	This publication	KT257642
AMDV-Jilin8	2014	China, Jilin	China	This publication	KT257643
AMDV-Jilin9	2014	China, Jilin	China	This publication	KT257644
AMDV-Jilin13	2014	China, Jilin	China	This publication	KT257637
AMDV-Jilin15	2014	China, Jilin	China	This publication	KT257638
AMDV-Jilin18	2014	China, Jilin	China	This publication	KT257639
AMDV-Jilin19	2014	China, Jilin	China	This publication	KT257640
AMDV-LN1	2009	China, Liaoning	China	[13]	GU183264
AMDV-LN2	2009	China, Liaoning	China	[13]	GU183265
AMDV-LN3	2009	China, Liaoning	China	[13]	GU269892
AMDV-G	Late 1970s	USA	USA	[9]	M20036
AMDV-Utah	Late 1970s	USA	USA	[9]	Z18276
AMDV-Utah1-kit	1995	USA	USA	[3]	U39015
AMDV-TR	1995	USA	USA	[3]	U39013
AMDV-Far East	2003	Russia	Russia	Martynenko et al. Direct Submission	AY428961
ADV-Far East	2006	Russia	Russia	Martynenko Direct Submission	DQ371395
AMDV-Rus09	2014	Russia	Russia	Yatsentyuk Direct Submission	KJ174162
V9	2006	Ireland	Ireland	Jahns et al. Direct Submission	DQ630716
V13	2006	Ireland	Ireland	Jahns et al. Direct Submission	DQ630717
FIN05/C8	2009	Finland	Finland	Knuuttila et al. Direct Submission	GQ336866
AMDV-SL3	Early 1980s	Germany	Germany	Schuierer et al. Direct Submission	X97629

## Supplementary information

**Additional file 1: Fig. S1.** Phylogenetic tree of 37 AMDV isolates based on alignment of the partial fragments of VP2 gene using maximum parsimony method. Bootstrap values higher than 40% are shown (1000 replications). The reference sequences were obtained from the GenBank database, and are marked by the country origins (China, United States, Russia, Germany, Finland and Ireland). The strains were separated into groups A, B and C. One single branch that is a little different from neighbor-joining and maximum likelihood phylogenies, is marked by A′. Scale bar indicates the average number of amino acids substitutions per site.

## Data Availability

The sequences of all the strains generated or analysed during the current study are available in the GenBank repository [https://www.ncbi.nlm.nih.gov/nuccore], the accession numbers have been provided in Table 2.

## Abbreviations

AMDV: Aleutian mink disease parvovirus
AMD: Aleutian mink disease
PCR: Polymerase Chain Reaction
