# Supplementary figures and images for: Molecular epidemiology of Aleutian mink disease virus from fecal swab of mink in northeast China
Source: BMC Microbiol. 2020 Aug 1;20:234. doi: 10.1186/s12866-020-01910-8 (PMC7395569; doi:10.1186/s12866-020-01910-8)

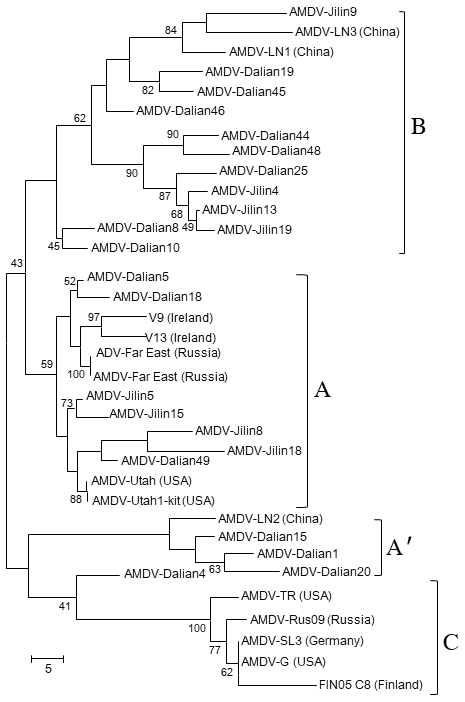

Supplement: Supplementary file 1 — Additional file 1: Fig. S1. Phylogenetic tree of 37 AMDV isolates based on alignment of the partial fragments of VP2 gene using maximum parsimony method. Bootstrap values higher than 40% are shown (1000 replications). The reference sequences were obtained from the GenBank database, and are marked by the country origins (China, United States, Russia, Germany, Finland and Ireland). The strains were separated into groups A, B and C. One single branch that is a little different from neighbor-joining and maximum likelihood phylogenies, is marked by A′. Scale bar indicates the average number of amino acids substitutions per site. [file 12866_2020_1910_MOESM1_ESM.tif]
